# Tenapanor for Irritable Bowel Syndrome With Constipation (IBS-C): A Systematic Review of Randomized Trials Assessing Food and Drug Administration (FDA) Composite Response, Durability, and Risk-of-Bias (RoB-2)

**DOI:** 10.7759/cureus.93337

**Published:** 2025-09-27

**Authors:** Abdulkreem Al-Juhani, Mahmoud S Desoky, Marwah Nasir Ahmad, Abdulrahman Alharthi, Rawiyah A Alkabkabi, Lujain Suhaqi, Jana H Alzahrani, Taif A Alotibi, Fatimah Almadih, Rodan Desoky

**Affiliations:** 1 Forensic Medicine, Forensic Medicine Center, Jeddah, SAU; 2 Surgery, King Abdulaziz University Faculty of Medicine, Jeddah, SAU; 3 Internal Medicine, Gastroenterology, Sultan Bin Abdulaziz Humanitarian City, Riyadh, SAU; 4 Medicine, Batterjee Medical College, Jeddah, SAU; 5 Medicine and Surgery, College of Medicine, Umm Al-Qura University, Makkah, SAU; 6 Medicine, Umm Al-Qura University, Makkah, SAU; 7 Medicine, Jazan University, Jazan, SAU; 8 Internal Medicine, Umm Al-Qura University, Makkah, SAU; 9 Medicine and Surgery, Alfaisal University College of Medicine, Riyadh, SAU

**Keywords:** ibs‑c, intestinal nhe3, randomized trials, systematic review, tenapanor

## Abstract

Tenapanor, a minimally absorbed inhibitor of intestinal Sodium/Hydrogen Exchanger 3 (NHE3), is approved for IBS with constipation (IBS‑C). We systematically reviewed randomized trials that used Food and Drug Administration (FDA)‑aligned weekly composite responders to consolidate the evidence on their efficacy, durability of benefit, and risk of bias. Following Preferred Reporting Items for Systematic Reviews and Meta-Analyses (PRISMA) 2020, we searched major databases and trial registries (January 2015 - August 2025) for adult, placebo‑controlled parallel‑group randomized controlled trials (RCTs) (≥12 weeks) of oral Tenapanor in IBS‑C. The primary endpoint was the FDA composite (≥30% abdominal pain reduction and ≥1 additional complete spontaneous bowel movement in the same week, sustained ≥6/12 or ≥13/26 weeks).

Patient‑reported outcomes were collected via daily e‑diaries. Risk of bias (RoB) was assessed with RoB-2; certainty with grading of recommendations, assessment, development, and evaluation (GRADE). Synthesis was narrative with supportive pooled estimates from the evidence profile. We found six studies that met our inclusion criteria: three double‑blind RCTs (one Phase 2b, two Phase 3) plus an open‑label extension and two post‑hoc pooled analyses. Across RCTs using the approved 50 mg twice‑daily dose, composite responder rates favored Tenapanor: Phase 2b 50.0% vs 23.6%; Tenapanor IBS-C Phase 3 clinical program (T3MPO)‑1 27.0% vs 18.7%; T3MPO‑2 36.5% vs 23.7% (placebo‑adjusted differences 8-26%). Durable response also favored Tenapanor (e.g., 9/12 weeks: 13.7% vs 3.3% and 18.4% vs 5.3%; 13/26 weeks: 35.5% vs 24.3%). Pooled estimates indicated a higher likelihood of response [composite relative risk (RR) 1.59, 95% confidence interval (CI) 1.33-1.90; abdominal‑pain responder RR 1.32, 95% CI 1.17-1.49] with minimal heterogeneity. Diarrhea was the principal adverse event (≈13-16%) and the most common reason for discontinuation; serious events were uncommon. Overall risk of bias was low to some concerns; certainty of evidence was moderate.

In conclusion, Tenapanor confers clinically meaningful, durable improvements in pain and bowel function for adults with IBS‑C, with predictable mechanism‑related diarrhea as the main tolerability trade‑off. Head‑to‑head trials versus other prosecretory agents and longer‑term pragmatic studies are priorities, but current evidence supports Tenapanor as a patient‑centered option within guideline‑directed care.

## Introduction and background

Irritable bowel syndrome (IBS) is a persistent functional gastrointestinal condition marked by irregular bowel movements and recurring abdominal pain [1]. Subtyping is generally determined by stool consistency during episodes of irregular bowel movements, encompassing IBS with diarrhea (IBS-D), IBS with constipation (IBS-C), and mixed-pattern IBS (IBS-M) [2]. 

In comparison to other kinds, patients with IBS-C frequently have more pronounced and widespread stomach discomfort, accompanied by sensations of fullness, cramping, bloating, and pain. 

The pathophysiology of IBS is complex and not fully understood, despite the involvement of several pathways [3]. Worldwide, IBS impacts between 7-21% of individuals, with IBS‑C constituting about one‑third of these instances. The condition significantly impairs quality of life, decreases workplace productivity, and elevates healthcare utilization [4]. Historically, management strategies for IBS-C have encompassed laxatives, dietary fiber, and stool softeners; however, the quality and consistency of evidence for these methods are inconsistent, and treatment satisfaction frequently remains inadequate [4]. 

Prescription medications-specifically the type-2 chloride channel activator lubiprostone and the guanylate cyclase-C agonists linaclotide and plecanatide-can enhance overall IBS-C symptoms in suitably chosen people [1]. 

Tenapanor, a pioneering minimally absorbed small-molecule inhibitor of the intestinal sodium/hydrogen exchanger isoform 3 (NHE3), has received approval from the Food and Drug Administration (FDA) for the treatment of IBS-C. NHE3 is localized in the apical membrane of enterocytes in the small intestine and colon, facilitating epithelial sodium absorption. Tenapanor enhances luminal salt and water by decreasing NHE3, resulting in softened stool and expedited intestinal transit [3,5]. Preclinical investigations indicate possible antinociceptive effects on visceral sensation. A placebo-controlled clinical trial for IBS-C was assessed once daily for Tenapanor over four weeks at dosages of 10, 30, and 100 mg, demonstrating an improvement in IBS-C symptoms [6]. 

In light of these data, we performed a comprehensive analysis of randomized controlled trials (RCTs) to consolidate the evidence for Tenapanor in IBS-C. We specifically analyzed efficacy utilizing FDA-recommended composite responder outcomes that encompass enhancements in stomach discomfort and bowel function, evaluated the durability of response, and examined methodological quality employing the Cochrane RoB-2 tool.

## Review

Methods

Protocol and Eligibility Criteria

This systematic review adhered to the Preferred Reporting Items for Systematic Reviews and Meta-Analyses (PRISMA) 2020 principles. We analyzed parallel-group RCTs with people (≥18 years) diagnosed with irritable IBS-C using Rome III or IV criteria. Eligible trials compared oral Tenapanor at any dose to a placebo. Trials had to have at least 12 weeks of treatment and report the FDA composite responder outcome (≥30% reduction in abdominal pain and ≥1 extra full spontaneous bowel movement per week, sustained for ≥6 of 12 or ≥13 of 26 weeks). Secondary outcomes included the duration of response, quality of life (QoL), individual symptom domains (pain, bloating, stool consistency, general alleviation), and safety outcomes. We omitted pediatric studies, non-IBS-C subgroups, post-hoc analyses that lacked primary randomized data, and non-comparative designs. 

Information Sources and Search Strategies

We searched PubMed, Embase, Cochrane CENTRAL, and ClinicalTrials from January 1, 2015, to August 20, 2025, supplemented by the EU Clinical Trials Register, WHO ICTRP, and key conference proceedings [Digestive Disease Week (DDW), American College of Gastroenterology (ACG), United European Gastroenterology Week (UEGW)]. Reference lists for suitable papers and reviews were also examined. There were no language restrictions throughout the search step. The PubMed technique combines controlled vocabulary and free text terms for "Tenapanor" (including synonyms such as RDX5791) AND "irritable bowel syndrome with constipation" OR "IBS-C," together with randomized trial and placebo filters. Search strategies for all databases will be included in the supplement. 

Study Selection

**Figure 1:**
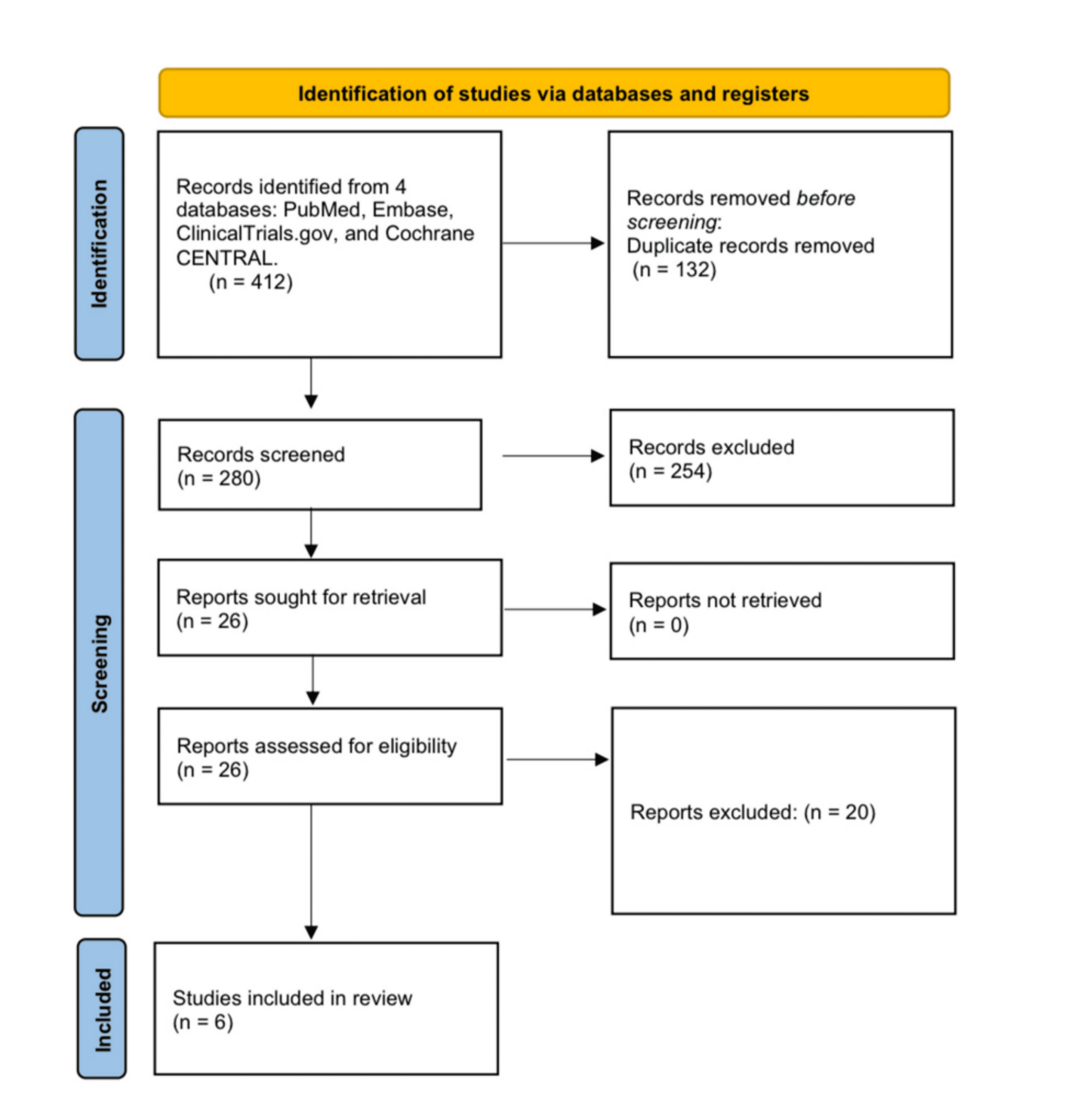
PRISMA Flow Diagram

Two reviewers individually screened titles/abstracts and then full texts based on predetermined criteria. The reasons for exclusion were documented. Disagreements were addressed through consensus or by a third reviewer. Full texts were prioritized above abstracts, and abstract-only entries were kept if no entire publication was available (Figure 1).

Data Extraction 

Data was extracted independently by two reviewers using a consistent template. The information extracted comprised the trial design, eligibility criteria, participant characteristics, intervention/comparator details, outcome definitions and timepoints, analysis population (ITT/mITT), funding source, and findings for prespecified outcomes. Wherever possible, missing information was sourced from trial registries or regulatory papers. 

Risk of Bias assessment

The Cochrane RoB-2 technique was used to assess risk of bias at the outcome level across five domains: randomization, variations from intended interventions, missing outcome data, outcome measurement, and selective reporting. Disagreements were settled through discussion. Detailed outcome-specific judgments are provided in Appendix 1.

Synthesis of Results

Because of the expected clinical and methodological heterogeneity, we did not conduct a statistical meta-analysis. Instead, we performed a narrative synthesis organized by outcome domain (primary efficacy, secondary efficacy, safety). The findings are given descriptively in text and tables, with a focus on the direction and degree of the effect, consistency across studies, and evidence quality. 

The certainty of evidence

The grading of recommendations, assessment, development, and evaluation (GRADE) framework was used to assess the reliability of evidence for the primary and key secondary outcomes. A table titled Summary of Findings summarizes the results.

Result

Selection and Features of Studies 

Six studies satisfied the inclusion criteria: three randomized, double-blind, placebo-controlled trials (one Phase 2b dose-finding and two Phase 3 trials) and three non-comparative/secondary analyses (one open-label extension and two post-hoc pooling analyses). In three randomized studies, persons with IBS-C (mostly women, mean age around 45 years) were recruited from U.S. multicenter locations and administered Tenapanor 50 mg bi-daily or a corresponding placebo for 12 weeks (Phase 2b; T3MPO-1) or 26 weeks (T3MPO-2). All trials recorded patient-reported outcomes by daily electronic or telephonic diaries, adhering to conventional validity criteria (≥4 days/week). The two pooled analyses utilized identical randomized datasets, whereas the open-label extension (T3MPO-3) monitored randomized completers on active Tenapanor for an additional 26-39 weeks to assess long-term safety (Table 1).

**Table 1:** Study Characteristics AS: abdominal score; BID: twice daily; ITT: intention-to-treat (analysis set); OLE: open-label extension;  RCT: randomized controlled trial; T3MPO: Tenapanor IBS-C Phase 3 clinical program.

Study	Trial/NCT	Country	Design	Rome Criteria	Arms (Dose)	N (Analysis Set)	Duration (Weeks)	Mean Age (y)	Women (%)
Chey et al. (Phase 2b) [1]	NCT01923428	USA	RCT, double‑blind, placebo‑controlled	Rome III	Tenapanor 50 mg BID vs Placebo	ITT: 84 / 89	12	45.7	86.8
Chey et al. (T3MPO‑1) [2]	NCT02621892	USA	Phase 3 RCT, double‑blind	Rome III	Tenapanor 50 mg BID vs Placebo	ITT: 307 / 299	12 (double‑blind) + 4‑week randomized withdrawal	45	81.4
Chey et al. (T3MPO‑2 [3])	NCT02686138	USA	Phase 3 RCT, double‑blind	Rome III	Tenapanor 50 mg BID vs Placebo	ITT: 293 / 300	26	45.4	82.1
Lembo et al. (T3MPO‑3 OLE)[4]	NCT02727751	USA	Open‑label extension (single‑arm)	Rome III in parent trials	Tenapanor 50 mg BID (all patients)	Safety set: 312 (single‑arm)	Up to +26–39 beyond RCT	Reported in full text	Reported in full text
Lembo et al. (Post‑hoc pooled AS )[5]	NCT01923428; NCT02621892; NCT02686138	USA (multicenter RCT datasets)	Post‑hoc pooled analysis of 3 RCTs	Rome III in parent trials	Tenapanor 50 mg BID vs Placebo (pooled)	Pooled ITT: 684 / 688	12 (from each RCT)	See parent RCTs	See parent RCTs
Brenner et al. (Post‑hoc subgroup) [6]	NCT02621892; NCT02686138	USA (multicenter RCT datasets)	Post‑hoc pooled subgroup analysis	Rome III in parent trials	Tenapanor 50 mg BID vs Placebo (pooled)	Parent RCT pooled (T3MPO‑1 + T3MPO‑2); subgroup Ns per analysis	12 (analysis window)	See parent RCTs	See parent RCTs

Efficacy Outcomes 

Primary composite endpoint responder (FDA 6/12 weeks). Tenapanor improved the FDA composite endpoint [≥30% reduction in abdominal pain and ≥1 additional complete spontaneous bowel movement (CSBM) within the same week] in all randomized trials, with absolute differences compared to placebo ranging from approximately eight to 26 percentage points. In the Phase 2b research (50 mg arm), the composite response rate was 50.0% compared to 23.6%. In T3MPO-1 (12 weeks), it was 27.0% versus 18.7%, and in T3MPO-2 (26 weeks), it was 36.5% versus 23.7%. These advancements were shown in elevated abdominal pain responder rates and enhancements in bowel frequency (CSBM responders). Durable response analyses also favored Tenapanor: in T3MPO-1, the proportion of 9/12-week composite responders was 13.7% compared to 3.3%; in T3MPO-2, the 9/12-week and 13/26-week composites were 18.4% versus 5.3% and 35.5% versus 24.3%, respectively.

Domains of abdominal symptoms. Subsequent composite, post-hoc pooled analyses utilizing the abdominal score [(AS); pain, discomfort, bloating] validated significant decreases with Tenapanor compared to placebo over 12 weeks, evidenced by least-squares mean change and increased proportions achieving AS response at six, 12, and nine weeks. A supplemental pooled analysis indicated that abdominal symptom relief occurs even in a strict no-CSBM subgroup (≥6 of the first 12 weeks without a CSBM), suggesting that analgesic and sensory effects may not be exclusively dependent on stool frequency (Table 2).

**Table 2:** Outcomes & Safety AE: adverse event; AS: abdominal score; ARR: absolute risk reduction; AS3: 3‑item abdominal score; CSBM: complete spontaneous bowel movement; FDA: Food and Drug Administration; LS: least squares; NNT: number‑needed‑to‑treat; OLE: open‑label extension; Pbo: placebo; RCT: randomized controlled trial; TEAE: treatment‑emergent adverse event; T3MPO: Tenapanor IBS-C Phase 3 clinical program. NNTs are approximate and conservatively calculated as the ceiling of 1/ARR based on reported responder percentages.

Study	Focal Endpoint (study‑appropriate)	Main Numeric Result	Effect Added Value	Safety Headline	Clinical Takeaway
Chey et al. (Phase 2b) [1]	FDA composite 6/12 (≥30% pain ↓ + ≥1 CSBM ↑ same week)	Ten vs Pbo: 50.0% vs 23.6% (p<0.001)	ARR 26.4% → NNT ≈ 4	Any AE 50.6% vs 42.2%; diarrhea 11.2% vs 0%; SAEs 0% vs 1.1%; discontinuations 4.5% vs 3.3%	Large incremental benefit with a mechanism‑consistent diarrhea signal; most events early and manageable.
Chey et al. (T3MPO‑1) [2]	FDA composite 6/12; durability 9/12	27.0% vs 18.7% (p=0.020); 9/12: 13.7% vs 3.3% (p<0.001)	ARR 8.3% → NNT ≈ 13	Diarrhea 14.6% vs 1.7%; treatment‑related diarrhea 13.3% vs 0.7%; discontinuations 7.4% vs 0.7%	Moderate effect size (conservative NNT) with predictable tolerability; monitoring in early weeks is prudent.
Chey et al. (T3MPO‑2) [3]	FDA composite 6/12 in a 26‑week trial; durability 13/26	36.5% vs 23.7% (p<0.001); 13/26: 35.5% vs 24.3% (p=0.003)	ARR 12.8% → NNT ≈ 8	Diarrhea ~16% (47/293); discontinuations due to diarrhea 6.5% vs 0.7%; SAEs uncommon; no deaths	Sustained benefit over 26 weeks (NNT≈8), balanced against a manageable diarrhea‑driven discontinuation rate.
Lembo et al. (T3MPO‑3 OLE) [4]	Long‑term tolerability on active therapy (single‑arm extension)	TEAEs 37.5%; diarrhea 10.6%; ≥52‑week subset TEAEs 57.8% (two severe diarrhea cases overall); no deaths	Added value: demonstrates persistence of tolerability signals with extended exposure beyond RCT windows.	Events predominantly mild/moderate; safety profile concordant with mechanism and RCT experience	Supports real‑world continuity of therapy while counselling on early diarrhea and long‑term monitoring.
Lembo et al. (Post‑hoc pooled AS) [5]	Abdominal Score (AS) over 12 weeks (pooled RCT data)	AS responder 6/12: 44.4% vs 32.4%; 9/12: 30.6% vs 20.5% (both p<0.0001); LS mean Δ −2.66 vs −2.09	ARR 12.0% → NNT ≈ 9	Safety consistent with parent RCTs; diarrhea is the most frequent AE with Tenapanor	Highlights symptom‑domain benefits (pain/bloating/discomfort) even when stool‑frequency responses vary.
Brenner et al. (Post‑hoc subgroup) [6]	AS3 (pain, bloating, discomfort) within stringent subgroups (e.g., no‑CSBM ≥6/12)	AS3 responder 6/12: 40.2% vs 29.6% (p=0.008); LS mean Δ −1.74 vs −1.29 (p=0.007)	ARR 10.6% → NNT ≈ 10	Safety mirrors parent RCTs; diarrhea remains the leading AE	Demonstrates abdominal‑symptom benefit even in subgroups with limited stool‑frequency change.

Safety and Tolerability 

Diarrhea, along with the drug's NHE3 action, was the predominant adverse event, typically occurring early and being self-limiting, and it was responsible for the majority of discontinuations in the active treatment groups. In T3MPO‑1, treatment-related diarrhea was observed in approximately 13% of people receiving Tenapanor, compared to less than 1% in the placebo group; in T3MPO‑2, diarrhea was reported in around 16% of Tenapanor recipients, resulting in discontinuation in 6.5% of cases, against 0.7% in the placebo group. Serious adverse events were infrequent and seldom associated with therapy; no fatalities were recorded in the randomized trials. The open-label extension indicated a safety profile aligned with the randomized controlled trials during prolonged exposure (total treatment-emergent adverse events around 38% in the complete open-label extension safety cohort and approximately 58% among those exposed for 52 weeks or more), with diarrhea persisting as the predominant occurrence (Table 2).

RoB-2 Across Randomized Trials

The risk of bias in T3MPO-1 was assessed as low across all areas, including strong centralized randomization and allocation, double blinding, prespecified analysis, and conservative management of missing weekly diaries. The Phase 2b study exhibited low risk in terms of randomization, measurement, and reporting; however, it raised certain concerns overall due to a modified intention-to-treat analysis that necessitated at least one valid week of diary data, resulting in the post-randomization removal of a small number of participants without early diary entries. T3MPO‑2 exhibited minimal risk in measurement and reporting; nonetheless, it raised concerns for two reasons: (i) insufficient detail about sequence creation and allocation concealment in the main text, and (ii) the exclusion of a small randomized cohort from one site following a recorded breach of good clinical practice (GCP) post-randomization. In all three RCTs, the absence of outcome data was reduced by the predetermined valid-week criterion (weeks with less than four diary days were classified as non-responses) and intra-week imputation methods. The outcome-specific RoB-2 assessments correspond with the operational characteristics detailed in the trial methodologies (Tables 3, 4).

**Table 3:** RoB-2 — Randomized Controlled Trials (Effect of Assignment; Primary Efficacy Outcome) CSBM: complete spontaneous bowel movement; FDA: Food and Drug Administration; GCP: good clinical practice; ITT: intention-to-treat (analysis set); IVRS: Interactive Voice Response System; IWRS: Interactive Web Response System; PRO: patient-reported outcome.

Study/ Trial ID	Outcome assessed	D1. Randomization process	D2. Deviations from intended interventions	D3. Missing outcome data	D4. Measurement of the outcome	D5. Selection of the reported result	Overall RoB-2
Chey et al. (Phase 2b) [1] NCT01923428	CSBM responder ≥1/wk for ≥6 of 12 weeks	Low — Computer‑generated IWRS; site‑level blocks; double‑blind.	Some concerns — mITT required ≥1 valid diary week; a few randomized excluded for no early diaries.	Low — <20% missing; weeks with <4 days counted as non‑response; within‑week imputation.	Low — PROs via IVRS under blinding; identical placebo.	Low — Registered (NCT01923428); prespecified endpoints reported.	Some concerns
Chey et al. (T3MPO‑1) [2] NCT02621892	FDA composite responder (6/12 weeks)	Low — Centralized IWRS with block randomization; double‑blind.	Low — ITT; analysis by assigned group; adherence monitored; no co‑intervention issues.	Low — ≈15% attrition; valid‑week rule (≥4 days) with conservative imputation as non‑response.	Low — PROs via IVRS in a blinded setting.	Low — Registered with prespecified hierarchy; results per SAP.	Low
Chey et al. (T3MPO‑2) [3] NCT02686138	FDA composite responder (6/12 weeks)	Some concerns — Randomized, blinded, but the main text lacks detail on sequence/allocation methods.	Some concerns — 27 randomized from 1 site were excluded after a GCP breach (post‑randomization exclusion).	Low — ~22% attrition; valid‑week rule; non‑response imputation for insufficient diary days.	Low — PROs via IVRS; blinding maintained.	Low — Registered; prespecified outcomes reported.	Some concerns

**Table 4:** ROBINS‑I — Nonrandomized/Post‑hoc/Extension Analyses (Primary Outcome Assessed in Each Study) AE: adverse event; AS: abdominal score; AS3: 3-item abdominal score (pain, bloating, discomfort); BID: twice daily; CSBM: complete spontaneous bowel movement; ITT: intention-to-treat (analysis set); IVRS: Interactive Voice Response System; OLE: open-label extension; PRO: patient-reported outcome; RCT: randomized controlled trial; ROBINS-I: risk of bias in non-randomized studies of interventions; SAE: serious adverse event; TEAE: treatment-emergent adverse event; T3MPO: Tenapanor IBS-C Phase 3 clinical program.

Study	Design/dataset	Outcome assessed	Confounding	Selection of participants	Classification of interventions	Deviations from intended interventions	Missing data	Measurement of outcomes	Selection of reported results	Overall ROBINS‑I
Lembo et al.(T3MPO‑3 OLE) [4]	Single‑arm, open‑label extension (50 mg b.i.d.)	Long‑term safety (TEAE/diarrhea incidence over up to 26–39 weeks)	Critical — No concurrent comparator; safety incidence cannot be causally attributed.	Serious — Entry required completion of prior RCT; susceptible to selection by tolerability/response.	Low — All participants received Tenapanor; exposure classification is clear.	Moderate — Open‑label; management may vary; adherence not blinded.	Moderate — Attrition over long follow‑up; differential discontinuation possible.	Moderate — Open‑label reporting of AEs may inflate detection.	Low — Preplanned safety reporting consistent with protocol.	Critical
Lembo et al.(AJG post‑hoc pooled AS) [5]	Post‑hoc pooled analysis of 3 RCTs (Phase 2b, T3MPO‑1, T3MPO‑2)	Abdominal Score change and responder (6/12; 9/12) vs placebo	Moderate — Parent trials randomized, but post‑hoc pooling and analytic choices may introduce imbalance.	Moderate — Uses analysis populations across studies; inclusion depends on diary completeness.	Low — Randomized assignment preserved from parent RCTs.	Low — Based on blinded RCT data; analyses by assigned group.	Low — Valid‑week rules and ITT/mITT handling described in parent trials.	Low — PROs via IVRS; blinded during data collection.	Moderate — Post‑hoc endpoints increase selective reporting risk.	Moderate
Brenner et al.(Dig Dis post‑hoc) [6]	Post‑hoc pooled subgroup analysis (no‑CSBM subgroup, etc.)	AS3 change/responder at week 12 within subgroups	Serious — Subgroups defined by post‑randomization variables (weekly CSBM); risk of collider bias.	Serious — Inclusion by subgroup status after randomization; may differ by treatment.	Low — Intervention classification from parent RCTs.	Low — Based on blinded RCT conduct; effect of assignment retained.	Moderate — Subgroup analyses sensitive to diary completeness.	Low — PROs via IVRS; blinded during RCTs.	Moderate — Post‑hoc focus and multiple comparisons raise selective reporting concerns.	Serious

Certainty of Evidence (GRADE)

The certainty of evidence was moderate for both prespecified outcomes throughout the three randomized, double-blind trials. The pooled impact for the FDA composite responder (≥6/12 weeks) was relative risk (RR) 1.59 (95% CI 1.33-1.90) with minimal heterogeneity (I² = 7.5%), indicating an absolute increase of +127 per 1,000 treated (ranging from +72 to +193; baseline placebo risk 215/1,000). Certainty was reduced by one level for risk of bias due to modified intention-to-treat (ITT) exclusions in the Phase 2b trial and a post-randomization site exclusion in T3MPO-2; there were no reductions for inconsistency, imprecision, or indirectness. The pooled effect for the abdominal-pain responder (≥6/12 weeks) was RR 1.32 [95% confidence interval (CI) 1.17-1.49] with minimal heterogeneity (I² ≈ 0%), resulting in an absolute increase of +120 per 1,000 (ranging from +64 to +184; baseline placebo risk 374/1,000). Publication bias was not officially evaluated due to the limited number of trials (k = 3). The open-label extension and post-hoc analyses were excluded from GRADE and are reported narratively. Refer to the GRADE Summary of Findings and Evidence Profile (Tables 5, 6).

**Table 5:** Summary of Findings (SoF) — Tenapanor vs Placebo (RCTs) FDA: Food and Drug Administration; T3MPO: Tenapanor IBS-C Phase 3 clinical program. Certainty starts at HIGH for RCTs and was downgraded one level for risk of bias (Phase 2b mITT exclusions; T3MPO‑2 post‑randomization site exclusion; limited allocation‑concealment detail). No downgrade for inconsistency (I² ≈ 7.5% for FDA composite; 0% for pain), imprecision (CIs exclude null), or indirectness.

Outcome (follow‑up)	№ participants (studies)	Contributing RCTs (first author)	Relative effect (RR, 95% CI)	Anticipated absolute effects (per 1,000)	Certainty (GRADE)	Plain‑language summary
FDA composite responder (≥6/12 weeks)	1372 (3 RCTs)	Chey et al. [1] ; Chey et al. [2] (T3MPO‑1); Chey et al. [3] (T3MPO‑2)	RR 1.59 (1.33–1.90)	Placebo: 215/1,000, Tenapanor: 342/1,000 (287–408), Absolute difference: +127/1,000 (72–193).	MODERATE ⬤⬤⬤◯	Tenapanor likely increases the probability of achieving the FDA composite response over 12 weeks.
Abdominal pain responder (≥6/12 weeks)	1372 (3 RCTs)	Chey 2017 [1] ; Chey 2020 [2] (T3MPO‑1); Chey 2021 [3] (T3MPO‑2)	RR 1.32 (1.17–1.49)	Placebo: 374/1,000, Tenapanor: 494/1,000 (438–558), Absolute difference: +120/1,000 (64–184)	MODERATE ⬤⬤⬤◯	Tenapanor likely increases the chance of meaningful abdominal pain improvement over 12 weeks.

**Table 6:** GRADE Evidence Profile — Domain Judgments by Outcome (With Contributing First Authors) FDA: Food and Drug Administration; T3MPO: Tenapanor IBS-C Phase 3 clinical program.

Outcome	№ studies (design)	Contributing RCTs (first author, year)	Risk of bias	Inconsistency	Indirectness	Imprecision	Publication bias	Overall certainty (GRADE)	Effect (RR, 95% CI)	Absolute effect (per 1,000)	Comments
FDA composite responder (≥6/12 weeks)	3 (RCTs)	Chey et al. [1]; Chey et al. [2](T3MPO‑1); Chey et al. [3](T3MPO‑2)	Downgraded (some concerns in Phase 2b and T3MPO‑2)	No downgrade (I² ≈ 7.5%)	No downgrade (direct population and outcomes)	No downgrade (95% CI excludes null)	No downgrade (not suspected; small k)	MODERATE ⬤⬤⬤◯	RR 1.59 (1.33–1.90)	Placebo 215/1,000 → Tenapanor 342/1,000 (+127/1,000; 287–408)	Consistent direction of effect across trials; absolute gain ≈ 127 per 1,000 treated.
Abdominal pain responder (≥6/12 weeks)	3 (RCTs)	Chey et al. [1] ; Chey et al. [2](T3MPO‑1); Chey et al. [3](T3MPO‑2)	Downgraded (same reasons as above)	No downgrade (I² ≈ 0.0%)	No downgrade	No downgrade	No downgrade	MODERATE ⬤⬤⬤◯	RR 1.32 (1.17–1.49)	Placebo 374/1,000 → Tenapanor 494/1,000 (+120/1,000; 438–558)	Effect consistent and clinically relevant; absolute gain ≈ 120 per 1,000 treated.

Overall Synthesis 

The study features collectively suggest a homogeneous cohort (Rome III IBS-C, primarily female, with a mean age in the mid-40s) and a uniform intervention (Tenapanor 50 mg administered bi-daily). Tenapanor demonstrated an enhancement in the FDA composite responder rate across all randomized comparisons, with corroborated benefits in stomach pain and the frequency of CSBM. Moreover, the persistence of response was noted at more stringent thresholds. The safety level was adequate, and the mechanism was reliable, with diarrhea as the main tolerability compromise and a restricted incidence of side events.

Discussion 

Principal Findings in Context

In the present analysis, improvements in abdominal symptom burden were observed even when changes in bowel frequency were modest, echoing observations that Tenapanor can reduce pain, discomfort, and bloating beyond stool metrics alone [6]. The consistency of benefit across the development program strengthens this signal: efficacy in Phase 2 IBS‑C [7], durable effects in the 12‑week T3MPO‑1 trial [8], sustained response through 26 weeks in T3MPO‑2 [9], reassurance from ≥52‑week safety data [10], and targeted reductions in abdominal symptoms in a post‑hoc analysis [11].

Position Within Guidance and Symptom Frameworks

Current US practice recommendations place secretagogues and NHE3 inhibition among evidence‑based options for moderate-severe IBS‑C, prioritizing global response and abdominal pain reduction as core outcomes [12]. The American Gastroenterological Association (AGA) guideline focusing on pharmacological management of IBS‑C similarly emphasizes treatments with reproducible effects on both bowel and abdominal symptoms [13]. A network meta‑analysis comparing secretagogues showed class‑wide superiority over placebo while highlighting differences across symptom domains that can guide individualized choice [14]. Our endpoint strategy was anchored in Rome IV criteria and the disorders‑of‑gut-brain interaction framework, in which abdominal pain remains the defining feature of IBS [15,16].

Accounting for Placebo and Trial Design

Placebo responses complicate IBS trials and demand rigorous outcome definitions. Earlier meta‑analyses indicate substantial placebo rates for global improvement and pain [17,18], and more recent syntheses suggest that trial features (e.g., dosing frequency and run‑in) modulate these rates [19]. When restricted to modern FDA‑aligned endpoints in trials of licensed agents, placebo response rates remain clinically meaningful yet lower for composite responders over ≥9 of 12 weeks, underscoring the value of stringent definitions used in our study [20].

Comparative Efficacy Considerations

Our findings should be interpreted alongside data for other approved agents. Linaclotide demonstrated durable 26‑week improvements in IBS‑C [21] with confirmatory prespecified analyses across Phase 3 trials [22]. Lubiprostone improved both bowel and abdominal symptoms in two Phase‑3 RCTs [23], and plecanatide achieved consistent composite and sustained‑efficacy responses in parallel Phase‑3 studies [24]. While head‑to‑head comparisons are lacking, quantitative syntheses suggest broadly similar global efficacy across secretagogues with possible differences in specific symptom profiles such as bloating [14].

Mechanistic Interpretation: Analgesia and Barrier Biology

Beyond fluid secretion, the guanylate cyclase‑C (GC‑C)/cGMP axis has peripherally mediated analgesic effects that can attenuate colorectal nociceptor excitability and visceral hypersensitivity [25-27]. In preclinical and translational models, plecanatide‑class agonism also supports epithelial barrier function, offering a plausible route by which luminal signaling can modulate pain and inflammation‑linked sensitivity [28].

Why Sodium Transport Matters

Tenapanor targets the apical Na^+/H^+ exchanger NHE3, a key transporter for intestinal sodium absorption [29]. By inhibiting NHE3, Tenapanor reduces transcellular sodium uptake, increases luminal water, and alters stool hydration; preclinical work and human data corroborate the mechanistic specificity of this effect [30]. The capacity of Tenapanor to reduce intestinal phosphate absorption in clinical settings further confirms its epithelial mechanism of action, independent of systemic exposure [31]. Together, these observations provide a biologically coherent rationale for improvements in abdominal symptoms that need not track one‑for‑one with stool frequency.

Disease Burden and Phenotype Heterogeneity

Globally, IBS affects large populations and imposes substantial quality‑of‑life and health‑care burdens [32]. Rome IV populations tend to have more severe longitudinal trajectories than Rome III cohorts, with higher health‑care utilization and greater persistent pain-a context in which therapies with meaningful abdominal‑symptom effects can be particularly valuable [33]. Systems‑level models of IBS underscore that gut‑brain, immune, epithelial, and microbial factors interact to shape abdominal pain and bloating, reinforcing the need for multimodal strategies [34].

Dietary Co‑management

Low-fermentable oligo-, di-, monosaccharides and polyols (FODMAP) interventions reduce IBS symptom severity in controlled trials [35,36]. In practice, diet and pharmacotherapy are not mutually exclusive: structured dietary support can be layered with pharmacologic therapy to address both luminal triggers and visceral sensitivity, while monitoring nutrition, microbiota, and patient preference.

Patient‑Reported Outcomes (PROs) and Digital Capture

Our use of patient‑reported measures aligns with validated IBS instruments and modern expectations for trials in disorders of gut-brain interaction. The IBS‑QOL instrument captures broad disease impact [37], and the IBS‑SSS provides a responsive symptom index with established thresholds for clinically important change [38]. Modern smartphone end‑of‑day diaries can support high adherence and granular, low‑recall‑bias collection of abdominal symptoms and bowel outcomes over time, enhancing outcome reliability in both trials and practice [39].

Methodologic Rigor and Certainty

Risk of bias was minimized using contemporary approaches and should be appraised formally with RoB-2 [40], while the overall certainty of evidence and strength of recommendations are best articulated using GRADE [41]. A full breakdown for each randomized trial is available in Appendix 1, and corresponding non-randomized/post-hoc assessments are shown in Appendix 2. These frameworks enable transparent translation of trial signals into clinical guidance.

Clinical Implications and Future Work

Taken together, our data, prior Tenapanor trials, and the broader evidence base suggest that epithelial‑targeted therapies can deliver clinically meaningful improvements in abdominal pain and bloating in IBS-C, not solely via laxation but also through peripheral analgesic and barrier‑modulating mechanisms [42]. This aligns with contemporary BSG and North American guidance that advocates individualized care combining diet, centrally acting and peripherally acting agents, and psychological strategies where appropriate [43]. Continued bench‑to‑bedside work on GC‑C/cGMP biology and epithelial transport (including GC‑C agonists) may yield further ways to modulate pain pathways and epithelial homeostasis in IBS‑C [44].

## Conclusions

Tenapanor represents a significant progression in the treatment of IBS-C, delivering consistent enhancements in abdominal discomfort and bowel function, with a tolerability profile predominantly influenced by mechanism-related diarrhea. Although its advantages are corroborated by moderate-certainty evidence, the lack of head-to-head trials and insufficient data on long-term effects underscore the necessity for additional research. 

In clinical practice, Tenapanor is regarded as an effective, patient-centered alternative within the wider therapeutic framework, particularly when treatment objectives prioritize sustained alleviation of pain and gastrointestinal symptoms.

## Appendices

Appendix 1 

**Table 7:** RoB 2 — Randomized Controlled Trials (Effect of Assignment; Primary Efficacy Outcome) CSBM: complete spontaneous bowel movement; FDA: Food and Drug Administration; ITT: intention-to-treat (analysis set); IVRS: Interactive Voice Response System; IWRS: Interactive Web Response System; PRO: patient-reported outcome; T3MPO: Tenapanor IBS-C Phase 3 clinical program.

Study / Trial ID	Outcome assessed	D1. Randomization process	D2. Deviations from intended interventions	D3. Missing outcome data	D4. Measurement of the outcome	D5. Selection of the reported result	Overall RoB 2
Chey et al. [1] (Phase 2b) NCT01923428	CSBM responder ≥1/wk for ≥6 of 12 weeks	Low — Computer‑generated IWRS; site‑level blocks; double‑blind.	Some concerns — mITT required ≥1 valid diary week; a few randomized excluded for no early diaries.	Low — <20% missing; weeks with <4 days counted as non‑response; within‑week imputation.	Low — PROs via IVRS under blinding; identical placebo.	Low — Registered (NCT01923428); prespecified endpoints reported.	Some concerns
Chey et al. [2] (T3MPO‑1) NCT02621892	FDA composite responder (6/12 weeks)	Low — Centralized IWRS with block randomization; double‑blind.	Low — ITT; analysis by assigned group; adherence monitored; no co‑intervention issues.	Low — ≈15% attrition; valid‑week rule (≥4 days) with conservative imputation as non‑response.	Low — PROs via IVRS in blinded setting.	Low — Registered with prespecified hierarchy; results per SAP.	Low
Chey et al. [3] (T3MPO‑2) NCT02686138	FDA composite responder (6/12 weeks)	Some concerns — Randomized, blinded, but main text lacks detail on sequence/allocation methods.	Some concerns — 27 randomized from 1 site excluded after a GCP breach (post‑randomization exclusion).	Low — ~22% attrition; valid‑week rule; non‑response imputation for insufficient diary days.	Low — PROs via IVRS; blinding maintained.	Low — Registered; prespecified outcomes reported.	Some concerns

Appendix 2 

**Table 8:** ROBINS‑I — Nonrandomized/Post‑hoc/Extension Analyses (Primary Outcome Assessed in Each Study) AE: adverse event; AS: Abdominal Score; AS3: 3-item Abdominal Score (pain, bloating, discomfort); CSBM: complete spontaneous bowel movement; ITT: intention-to-treat (analysis set); VRS: Interactive Voice Response System; OLE: open-label extension; PRO: patient-reported outcome; RCT: randomized controlled trial; ROBINS-I: risk of bias in non-randomized studies of interventions; TEAE: treatment-emergent adverse event; T3MPO: Tenapanor IBS-C Phase 3 clinical program.

Study	Design / dataset	Outcome assessed	Confounding	Selection of participants	Classification of interventions	Deviations from intended interventions	Missing data	Measurement of outcomes	Selection of reported result	Overall ROBINS‑I
Lembo et al. [4] (T3MPO‑3 OLE)	Single‑arm, open‑label extension (50 mg b.i.d.)	Long‑term safety (TEAE/diarrhea incidence over up to 26–39 weeks)	Critical — No concurrent comparator; safety incidence cannot be causally attributed.	Serious — Entry required completion of prior RCT; susceptible to selection by tolerability/response.	Low — All participants received Tenapanor; exposure classification is clear.	Moderate — Open‑label; management may vary; adherence not blinded.	Moderate — Attrition over long follow‑up; differential discontinuation possible.	Moderate — Open‑label reporting of AEs may inflate detection.	Low — Preplanned safety reporting consistent with protocol.	Critical
Lembo et al. [5] (AJG post‑hoc pooled AS)	Post‑hoc pooled analysis of 3 RCTs (Phase 2b, T3MPO‑1, T3MPO‑2)	Abdominal Score change and responder (6/12; 9/12) vs placebo	Moderate — Parent trials randomized, but post‑hoc pooling and analytic choices may introduce imbalance.	Moderate — Uses analysis populations across studies; inclusion depends on diary completeness.	Low — Randomized assignment preserved from parent RCTs.	Low — Based on blinded RCT data; analyses by assigned group.	Low — Valid‑week rules and ITT/mITT handling described in parent trials.	Low — PROs via IVRS; blinded during data collection.	Moderate — Post‑hoc endpoints increase selective reporting risk.	Moderate
Brenner et al. [6] (Dig Dis post‑hoc)	Post‑hoc pooled subgroup analysis (no‑CSBM subgroup, etc.)	AS3 change/responder at week 12 within subgroups	Serious — Subgroups defined by post‑randomization variables (weekly CSBM); risk of collider bias.	Serious — Inclusion by subgroup status after randomization; may differ by treatment.	Low — Intervention classification from parent RCTs.	Low — Based on blinded RCT conduct; effect of assignment retained.	Moderate — Subgroup analyses sensitive to diary completeness.	Low — PROs via IVRS; blinded during RCTs.	Moderate — Post‑hoc focus and multiple comparisons raise selective reporting concerns.	Serious
